# Transcripts derived from AmnSINE1 repetitive sequences are depleted in the cortex of autism spectrum disorder patients

**DOI:** 10.3389/fbinf.2025.1532981

**Published:** 2025-04-09

**Authors:** Nicolina Sciaraffa, Daniele Santoni, Andrea Li Greci, Swonild Ilenia Genovese, Claudia Coronnello, Walter Arancio

**Affiliations:** ^1^ Advanced Data Analysis Group, Ri. MED Foundation, Palermo, Italy; ^2^ Institute for System Analysis and Computer Science “Antonio Ruberti”, National Research Council of Italy (IASI-CNR), Rome, Italy; ^3^ Independent Researcher, Palermo, Italy; ^4^ Institute for Biomedical Research and Innovation, National Research Council of Italy (IRIB-CNR), Palermo, Italy

**Keywords:** autism spectrum disorder, repetitive sequences, neurogenesis, microRNA, embryonic development, autistic disorder, nervous system

## Abstract

**Aims:**

Autism spectrum disorder (ASD) is a brain developmental disability with a not-fully clarified etiogenesis. Current ASD research largely focuses on coding regions of the genome, but up to date much less is known about the contribution of non-coding elements to ASD risk. The non-coding genome is largely made of DNA repetitive sequences (RS). Although RS were considered slightly more than “junk DNA”, today RS have a recognized role in almost every aspect of human biology, especially in developing human brain. Our aim was to test if RS transcription may play a role in ASD.

**Methods:**

Global RS transcription was firstly investigated in *postmortem* dorsolateral prefrontal cortex of 13 ASD patients and 39 matched controls. Results were validated in independent datasets.

**Results:**

AmnSINE1 was the only RS significantly downregulated in ASD specimens. The role of AmnSINE1 in ASD has been investigated at multiple levels, showing that the 1,416 genes containing AmnSINE1 are associated with nervous system development and autism susceptibility. This has been confirmed in a different experimental setting, such as in organoid models of the human cerebral cortex, harboring different ASD causative mutations. AmnSINE1 related genes are transcriptionally co-regulated and are involved not only in brain formation but can specifically be involved in ASD development. Looking for a possible direct role of AmnSINE1 non-coding transcripts in ASD, we report that AmnSINE1 transcripts may alter the miRNA regulatory landscape for genes involved in neurogenesis.

**Conclusion:**

Our findings provide preliminary evidence supporting a role for AmnSINE1 in ASD development.

## Introduction

Autism spectrum disorders (ASDs) include a group of heterogeneous neurological and behavioral developmental disorders that can occur in infancy or early childhood and are characterized by impairments in social communication skills and specific stereotypic or repetitive behaviors (Hodges et al., 2020). ASDs are often interlaced with other syndromes of genetic origin (e.g., Down, Turner or Williams syndromes) complicating the process of diagnosis and clinical evaluation (Genovese and Butler, 2023).

Autism affects approximately 2% of children, and its heritability is estimated to be greater than 70% (Genovese and Butler, 2023) however, its inheritance is complex and not fully understood because ASD seems to be a multifactorial disorder strongly influenced by environmental and epigenetic factors. To date, approximately one thousand genes have been associated with ASD, and many individuals with ASD carry chromosomal aberrations (Genovese and Butler, 2023). Overall, even if the involvement of genetics is established, our knowledge of ASD heritability is far from exhaustive. An incomplete explanation of the genetic factors that contribute to the development of ASD is often referred to as “ASD missing heritability” (Masini et al., 2020).

However, despite the great heterogeneity of ASD, the vast majority of genes and associated activities involve convergence of specific biological processes, such as Wnt signaling and chromatin remodeling during neurogenesis, and neuronal activity, especially synaptic function and neuronal cell adhesion (Geschwind and State, 2015). Interestingly, ASD genes are disproportionately overrepresented in the gene-poor heterochromatin sub-bands, suggesting that heterochromatic regions and their associated activities may play a role in ASD (McGuire et al., 2016). Indeed, current ASD research largely focuses on coding regions of the genome, which account for approximately 2% of the whole human genome; however, to date, much less is known about the contribution of noncoding elements to ASD risk, which are highly promising for enhancing or even completing our understanding of ASD (Dominguez-Alonso et al., 2023).

The noncoding human genome is largely composed of repetitive DNA sequences (RSs), which represent approximately half of the human genome overall. Currently, RSs play a recognized role in almost every aspect of human biology, from embryonic development to aging and cancer transformation (Arancio and Coronnello, 2021; 2022; Gorbunova et al., 2021; Mangiavacchi et al., 2021; Wang et al., 2023; Zhang et al., 2022).

RSs can play a role in the regulation of gene expression by multiple mechanisms. RSs can act as cis-regulatory modules by providing binding sites for transcription factors, modifying local chromatin structure, and influencing DNA methylation patterns. RSs can also regulate transcription in trans by producing RNA molecules that can act as molecular scaffolds that recruit chromatin modifiers to specific genomic regions, or function as miRNA decoys or sponges, sequestering miRNAs away from other RNAs and thereby alleviating repression (and thereby acting as competing endogenous RNAs or ceRNAs) (Salmena et al., 2011; Taft et al., 2010).

Moreover, RSs can trigger signal cascades such as the proinflammatory cGAS-STING (cyclic GMP-AMP synthase -stimulator of interferon genes) signaling mimicking retroviral particles (Mathavarajah and Dellaire, 2024).

RSs are intrinsically difficult to study and challenging to explore. Indeed, dedicated pipelines of analysis are needed to study RS biology, including their localization in the genome and their transcriptional activity. Each RS is present in thousands of copies per haploid human genome, and they are often embedded in introns or regulatory sequences or are clustered in heterochromatic regions. RSs are routinely divided into five main classes: i) ancient retroviruses with preserved long terminal repeats (LTRs) sparsely integrated in the genome: the human endogenous retrovirus (HERV) families are the most active in humans; ii) evolutionary remnants of inactive DNA transposons; iii) satellite repeats, which are tandem repeats that constitute the vast majority of centromeres and telomeres but are also present in great numbers in constitutive heterochromatic regions; iv) long interspersed nuclear elements (LINEs), which are non-LTR retrotransposons, the most active of which are those of the LINE-1 family that are still retrotransposition competent and active in humans; and v) small interspersed nuclear elements (SINEs), which are nonautonomous retrotransposons that can be divided into many subfamilies following their sequence and origin. Some SINEs are able to retrotranspose but they need to hijack the activity of other retroelements (Padeken et al., 2015).

Interestingly, RSs are specifically active in the developing and adult human brain, and somatic retrotransposition of RSs alters the genetic landscape of the human brain (Baillie et al., 2011). Indeed, DNA extracted from the cerebellum, frontal cortex, subventricular zone and dentate gyrus revealed hundreds of RS somatic insertions in each of the analyzed tissues (Kurnosov et al., 2015). LINE-1 retrotransposition alters the hippocampal genomic landscape, enabling memory formation; indeed, the adult human hippocampus is a genetic mosaic due to ubiquitous LINE-1 mosaicism in hippocampal neurons (Bachiller et al., 2017; Upton et al., 2015). The SINE-VNTR-Alu (SVA) elements have been suggested to be potential modulators of neuropeptide gene expression (Gianfrancesco et al., 2017). AmnSINE1, a member of the SINE family that is specific for amniota genomes, can play an important role in mammalian-specific brain development (Hirakawa et al., 2009; Sasaki et al., 2008) and is of particular interest in the research here presented.

Due to its role in the development of the adult human brain, RS deregulation has been suggested to be a potential player in many neurological diseases (Misiak et al., 2019). This is the case for Alu elements (the most common SINEs in humans) in neurodegenerative disorders, in which they seem to alter the activity of genes involved in mitochondrial function (Larsen et al., 2018); alternatively, there is evidence that RSs are hypermethylated in multiple sclerosis patients (Neven et al., 2016).

A possible role for Alu sequences and LINE-1 elements has been proposed (Saeliw et al., 2022; 2023; Shpyleva et al., 2018) together with satellite sequences (Mitra et al., 2021) and HERVs (Balestrieri et al., 2012).

The aim of this work was to investigate the role of DNA repetitive sequences, specifically AmnSINE1, in ASD development. To this end, RS transcription in the *postmortem* dorsolateral prefrontal cortex of ASD patients and matched controls was investigated using RNA-seq datasets obtained from the European Nucleotide Archive (Wright et al., 2017) *via* our custom-developed analysis pipeline (Arancio, 2019). The study successfully identified AmnSINE1 as significantly downregulated in ASD specimens and explored its implications at multiple levels, including its association with genes related to nervous system development and autism susceptibility. In particular, the investigation focused on the association of AmnSINE1 with the expression of genes containing this element and its biological functions as noncoding RNAs. The role of these genes in single-cell data obtained from a public repository was transversally validated (Paulsen et al., 2022). Finally, the study examined the possible impact of AmnSINE1 on the miRNA regulatory landscape for genes involved in neurogenesis. Overall, the findings highlight a possible role of AmnSINE1 in ASD development.

## Materials and methods

### Datasets

In order to analyze repetitive sequences, sequencing libraries must be prepared by using Ribosomal RNA depletion (RiboZero Gold or similar). Poly-A enriched libraries should not be used to quantify the transcription from RS. Therefore, we performed extensive research on database repositories like Gene Expression Omnibus and European Nucleotide Archive using the following words: Autism and (RiboZero or TruSeq). After filtering for *Homo Sapiens* samples, three datasets were identified as suitable: GSE102741, GSE64018, and GSE76852. However, the GSE64018 was not immediately available (Irimia et al., 2014) and GSE76852 lacked ASD samples. Consequently, the primary dataset utilized for this research is GSE102741, which comprises data from the *postmortem* dorsolateral prefrontal cortex of 13 ASD patients and 39 matched controls (Wright et al., 2017). The associated bioproject PRJNA398545 allowed access to raw FASTQ files of transcriptomic experiments from strand-specific Ribosomal RNA depleted (RiboZero) library preparation and TruSeq RNA Sample Preparation v2 kit from Illumina. A manual search on Gene Expression Omnibus and European Nucleotide Archive yielded two additional datasets of interest. The first of these, bioproject PRJNA263196, involved the analysis of RNA-Seq for the corpus callosum sampled from the *postmortem* brain of 12 individuals, half of whom had been diagnosed with autism spectrum disorders. The RNA of each sample was subjected to RNA-seq library preparation with ScriptSeq™ Complete Gold Kit from Epicentre after Ribodepletion (Li et al., 2014). The second dataset, PRJNA869106, involved a comparative gene expression analysis of RNA-seq data from laser captured microdissection *postmortem* ASD Purkinje neurons compared to controls. The total RNA was prepared using the Nugen Ovation SoLo RNA-Seq Library Preparation Kit (Brandenburg et al., 2022).

### Analysis of RSs

The method of analysis was previously described in (Arancio, 2019). Here, a minor modification was used. In brief, the analyses were carried out within a Galaxy environment (Afgan et al., 2018; Jalili et al., 2020) at https://usegalaxy.org/. The FASTQ raw sequences were processed by the Trimmomatic tool (Galaxy Version 0.38) (Bolger et al., 2014) and quality checked. The Bowtie2 aligner (Galaxy Version 2.3.4.3) (Langmead and Salzberg, 2012) was used with very sensitive local parameters to retrieve the expression of RSs, oblivious of their genomic localization, and aligning the reads against pseudochromosomes containing the reference RS sequences. The same raw FASTQ data were aligned with the HISAT2 aligner (Galaxy Version 2.1.0) (Kim et al., 2015) using the Galaxy-embedded hg38 as a reference. The reads per gene were counted by featureCounts (Galaxy version 1.6.4) (Liao et al., 2014) using the genecode. v36. annotation.gtf annotation file.

### DEG analysis

The raw read counts of the RS and canonical genes were merged per sample. The following analysis was performed in R.

The differential expression analysis was performed with the DESeq2 tool (Love et al., 2014) using the following parameters: test = Wald, fitType = parametric, sfType = ratio, minReplicatesForReplace = 7, and betaPrior = TRUE. The correction of p-values for multiple comparisons was performed using the Benjamini–Hochberg method. The generation of the heatmap was achieved by employing the VST-transformed data, and the pheatmap function with the Euclidean distance and the complete clustering method.

### Identification of AmnSINE1-containing loci

The file containing the localization of AmnSINE1 was obtained from the UCSC Table Browser (Kent et al., 2002) and the list of genetic loci containing the sequence is reported in Supplementary Table S1.

### SFARI database

To identify genes that are known to be involved in autism, we employed the SFARI Gene database, an online resource for the autism research community that focuses on genes implicated in autism susceptibility (https://gene.sfari.org/; database version 2023).

### Gene ontology (GO) and disease enrichment analysis

In order to retrieve the activity in which the analyzed genes are involved, we performed both Gene Ontology (GO) and Disease Ontology (DO) enrichment analyses using the clusterProfiler R package. Given a vector of genes, this analysis returned the enrichment categories after FDR control.

### Identification of transcription factors

Transcription factor (TF) genes potentially involved in the regulation of AmnSINE1 expression were identified through the use of the TRANSPARENT python tool (Derelitto and Santoni, 2023). The analysis, based on a hypergeometric test, evaluated the enrichment of binding sites in the promoter regions of the genes of interest for each TF with respect to the background (all human genes). To ensure the reliability and robustness of the statistical results, a strict Bonferroni correction was applied to the p-values. Finally, the network of the selected TFs was presented using STRING (von Mering et al., 2005).

### Analysis of single-cell data

Paulsen and colleagues (Paulsen et al., 2022) used ASD organoid models of the human cerebral cortex to identify cell type-specific developmental abnormalities that result from haploinsufficiency in three ASD risk genes: *ARID1B*, *CHD8* and *SUV420H1.* The associated single-cell data were retrieved from https://singlecell.broadinstitute.org/single_cell/study/SCP1129/asd-mutated-brain-organoids. The pipeline used for preprocessing and cell annotation was described previously by Paulsen et al. (2022).

The present study focused on the analysis of cell lines and timepoints at which the authors identified a significant difference in cellular differentiation between mutant and wild-type organoids. Specifically, the analysis encompassed the HUESS66 cell line for *CDH8*-mutant organoids at the third month, and the Mito210 cell line for *ARID1B* and *SUV420H1* mutant organoids at the first month. The authors demonstrated that each mutation affects the development of two cortical neuronal lineages: GABAergic neurons and deep layer excitatory projection neurons.

Specifically, variations in cell type proportions and pseudotime values between control and mutant organoids have been demonstrated for newborn deep-layer projection neurons for the Mito210 cell line at the first month in the case of *ARID1B* and *SUV420H1* mutant organoids, and for 3-month-old GABAergic neurons from the HUESS66 cell line in the case of CDH8-mutant organoids.

In order to assess the DEGs, the Wilcoxon test with the Bonferroni correction was performed between mutant and control organoids twice. Initially, all cells for each organoid were considered, and, subsequently, the subset of the cell type of interest (i.e., GABAergic neurons and deep layer excitatory projection neurons) was considered.

The list of DEGs was intersected with those enriched in the AmnSINE1 cohort. A hypergeometric test was then employed to ascertain the statistical significance of the intersection. Gene Ontology (GO) and disease ontology enrichment analyses were performed as confirmatory tests.

### Competing endogenous RNA analysis

In order to analyze the competing endogenous RNA (ceRNA), three algorithms -PITA (Kertesz et al., 2007), miRanda (Enright et al., 2003) and TargetScan (Lewis et al., 2005)- were utilized to predict the miRNA that can bind AmnSINE1 transcripts. The following parameters were used to select the relevant miRNAs: i) miRanda: binding energy ≤−20 kcal/mol and score ≥140; ii) PITA: ΔΔE ≤−10 kcal/mol; and iii) TargetScan: binding site type = 8mer-1a, 7mer-1a or 7mer-m8. The prediction identified 451 potential miRNAs (Supplementary Table S2).

Second, all the genes potentially regulated by the 17 miRNAs predicted by all the algorithms were retrieved from the MBS database (Arancio et al., 2023), which uses the same parameters described above. Third, those containing AmnSINE1 were removed from these genes, and the remaining genes were ordered according to the number of putative regulatory miRNAs shared with AmnSINE1 (Supplementary Table S3). The genes regulated by at least 5 miRNAs were analyzed as described in the *Gene Ontology and disease enrichment analysis.*

## Results

### AmnSINE1 is downregulated in dorsolateral prefrontal cortex of ASD specimens

**FIGURE 1:**
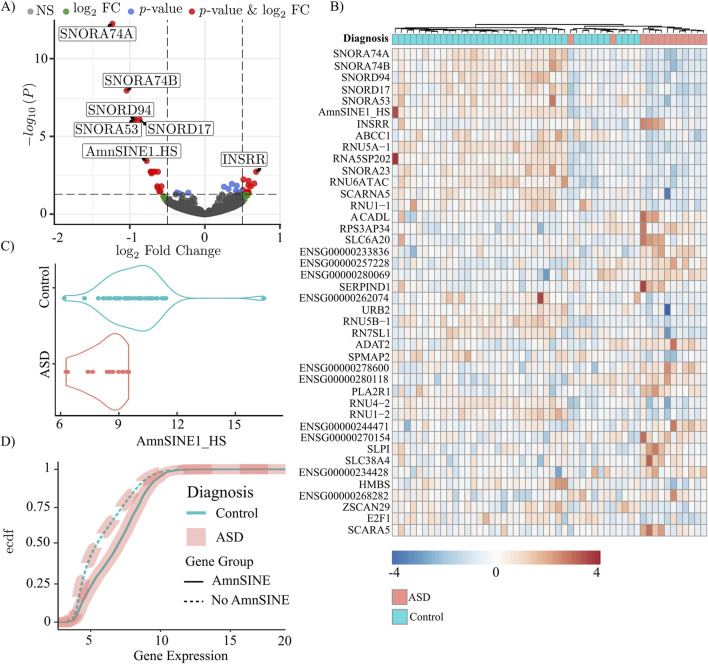
Genes and repetitive sequences in ASD (n = 13) and control (n = 39) specimens. In all panels, the light blue color represents the control, and the pink color represents the ASD samples. **(A)** Volcano plot of log2FC and adjusted p-values. The cutoff for fold change is >|0.5|, and the cutoff for p-value is 0.05. **(B)** Heatmap of the differentially expressed genes. The color scale indicates the normalized expression. **(C)** Violin plot of AmnSINE1 expression in ASD patients and controls. The difference was statistically significant, with a p-value = 0.00038. **(D)** Empirical cumulative distribution function (ecdf) of the expression of genes containing (solid line) and not containing (dashed) the AmnSINE1 sequence.

We investigated RS transcription in the *postmortem* dorsolateral prefrontal cortex of 13 ASD patients and 39 matched controls using the raw data published by Wright et al. (2017). The custom pipeline used for analysis retrieved the expression values of both the canonical genes and the RSs. The analysis yielded 42 entries, including both canonical genes and RSs, that were differentially expressed (adjusted p-value <0.05) (Figure 1A; Supplementary Table S4). Notably, among the differentially expressed genes, only one was a RS, AmnSINE1, and the remaining differentially expressed genes did not contain the AmnSINE1 sequence. Of the 42 differentially expressed genes, AmnSINE1 showed the third highest expression level, with base mean of 1,265 normalized reads, after the extremely highly expressed *RN7SL1* gene (the RNA component of the signal recognition particle 7SL1) at 497,261 reads and the *SCARNA5* gene (small Cajal body-specific RNA) at 3,048 reads. Consequently, our analyses focused on AmnSINE1 and its potential role in ASD. A heatmap depicting the normalized expression values of the DEGs revealed clear clustering of 11 out of the 13 ASD samples (Figure 1B). In particular, the DLPFC specimens were clustered into 2 subgroups: the first subgroup on the left, characterized by increased expression of AmnSINE1; the second subgroup, composed of ASD patients and a subgroup of nonaffected controls, was characterized by decreased expression of AmnSINE1. Specifically, the expression of AmnSINE1 was significantly downregulated in ASD patients (Figure 1C), with a logFC = −0.77 and a adjusted p-value = 0.00037.

Moreover, we found a significant positive correlation between AmnSINE1 and gene expression. The empirical cumulative distribution function revealed that genes containing the AmnSINE1 sequence exhibited significantly (p-value<10^−16^) higher levels of expression compared to other genes (Figure 1D). Conversely, the diagnostic factor (control/ASD) was not affected by the presence of AmnSINE1 (p-value = 0.7847).

### The quantification of RSs in the corpus callosum and purkinje cells of ASD specimens showed no significant differences

Using the same pipeline described above, we examined the RS transcription in the corpus callosum (Li et al., 2014) and in isolated human *postmortem* Purkinje neurons (Brandenburg et al., 2022). In both datasets, no significant differences in RS expression, and consequently in AmnSINE1, was found (Supplementary Figures S1A–C; Supplementary Table S4). However, also in these cases, we found a significant positive correlation between AmnSINE1 and gene expression. For both corpus callosum and Purkinje cells, genes containing the AmnSINE1 sequence exhibited significantly (p-value<10^−16^) higher levels of expression compared to other genes (Supplementary Figure S1B-D) and the diagnostic factor (control/ASD) was not affected by the presence of AmnSINE1 (p-value = 0.88 and p-value = 0.39 for corpus callosum and Purkinje cells, respectively).

### Loci containing AmnSINE1 are associated with ASD susceptibility and syndromic genes

**FIGURE 2:**
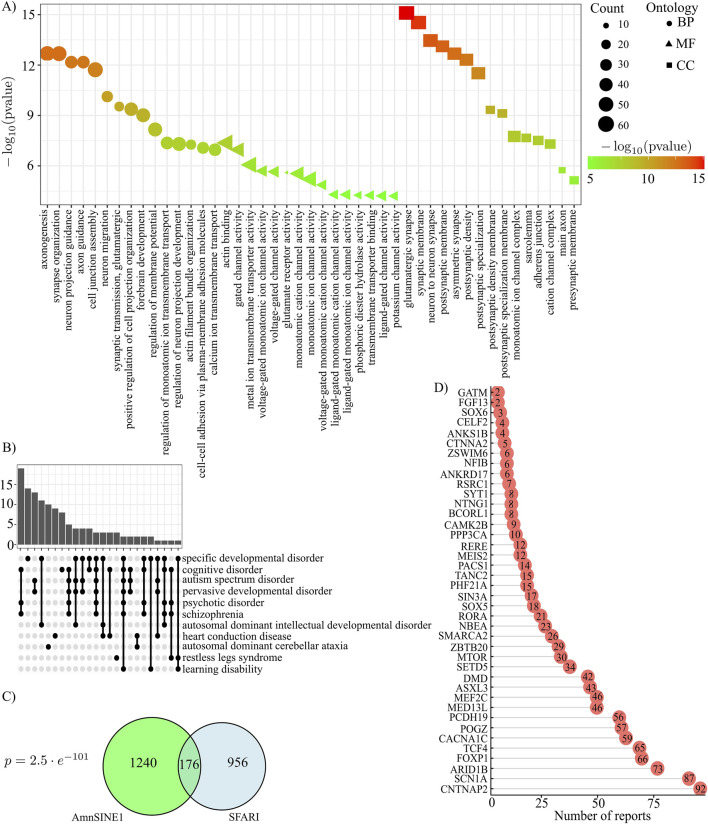
AmnSINE1-containing loci **(A)** Gene Ontology results in terms of biological processes, molecular functions and cellular components. The first 15 significant terms are represented. **(B)** Disease Ontology enrichment analysis results. **(C)** Venn diagram of AmnSINE1-containing loci and the SFARI database. There were 1132 SFARI genes in total since there was no Ensembl ID for 8 genes. The intersection was statistically significant (p-value = 2.5 ⨯ 10-101). **(D)** Bar plot of number of reports for 40 syndromic loci.

To analyze the association of AmnSINE1 with target genes in ASD, the initial step was to retrieve the genomic loci that contain AmnSINE1, yielding 1,416 loci (Supplementary Table S1). A gene ontology analysis clearly highlighted that these genes are enriched in nervous system terms (Figure 2A). Additionally, a disease enrichment analysis revealed an overrepresentation of these genes in cognitive disorders, autism spectrum disorders, and learning disability (Figure 2B). The comprehensive results of the gene ontology and disease enrichment analyses are reported in Supplementary Table S5.

Moreover, to identify genes that are known to be involved in autism, we intersected the list of genomic loci containing AmnSINE1 with the list of genes provided by the SFARI Gene database, a tool providing the autism research community with the most up-to-date information on all known human genes associated with ASD (Abrahams et al., 2013). At the time of the analysis, 1,140 genes were present in the database. Interestingly, 176 genes were found to be shared between the SFARI database and AmnSINE1-containing loci. The intersection of the two databases is statistically significant (p-value = 2.5 × 10^−101^) according to a hypergeometric test (Figure 2C). Among the 176 genes, 40 were classified as syndromic genes and the barplot in Figure 2D shows the number of actual publications demonstrating such evidence.

### A network of transcription factors (TFs) orchestrates the regulation of genes containing AmnSINE1 sequences, with HOX family exhibiting particular prominence

**FIGURE 3:**
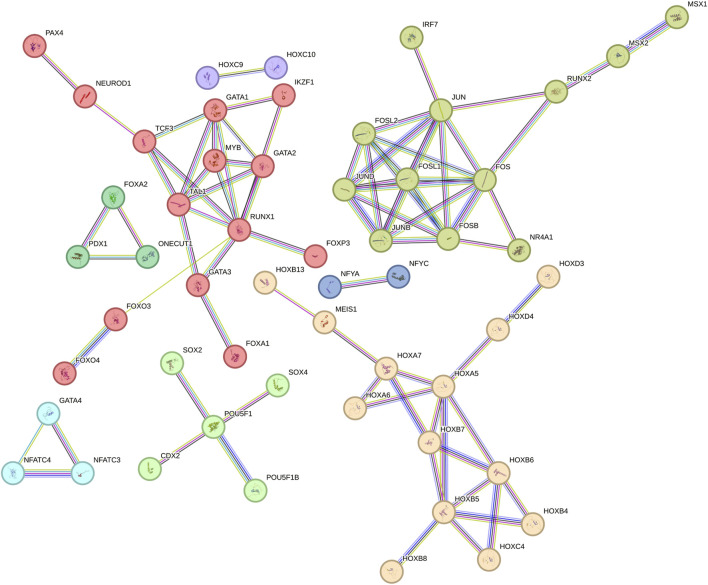
Network of transcription factors of AmnSINE1-containing genes obtained from STRING database.

To identify transcription factors (TFs) potentially involved in regulating the expression of genes containing AmnSINE1, the TRANSPARENT tool (Derelitto and Santoni, 2023) was used to detect TFs whose binding sites are enriched in the promoter regions of these genes (see Supplementary Table S6). The TFs identified in our analysis, along with their predicted interactions as obtained from the STRING database, are illustrated in Figure 3. In constructing the network, only interactions with a confidence score of at least 0.9 were included, ensuring that the resulting interactome reliably reflects high-confidence associations among the TFs, with 82 interactions meeting this stringent criterion.

Notably, the identified TFs exhibit a significantly higher degree of interconnectivity than would be expected by chance, as evidenced by a protein-protein interaction (PPI) enrichment p-value of less than 10^−6^. Therefore, it can be concluded that these TFs share biologically meaningful relationships, which reinforces the functional coherence of the identified TF set. Additionally, we applied a network-based approach to delineate clusters of TFs potentially involved in specific biological functions. Such clusters were identified based on the connections in the network, as defined by the interactions annotated in the STRING database. To facilitate further discussion of the role of specific subclusters, single subclusters are highlighted with different colors. Among the clusters with higher number of nodes there is the HOX family, a pivotal contributor to brain development (Gonçalves et al., 2020). Additionally, other TFs implicated in neurodevelopment, such as NEUROD1 (Singh et al., 2022) and SOX2 (Wang et al., 2022), are also discernible within this network.

### Loci containing AmnSINE1 are differentially expressed in organoid models of ASD in the human cerebral cortex

**FIGURE 4:**
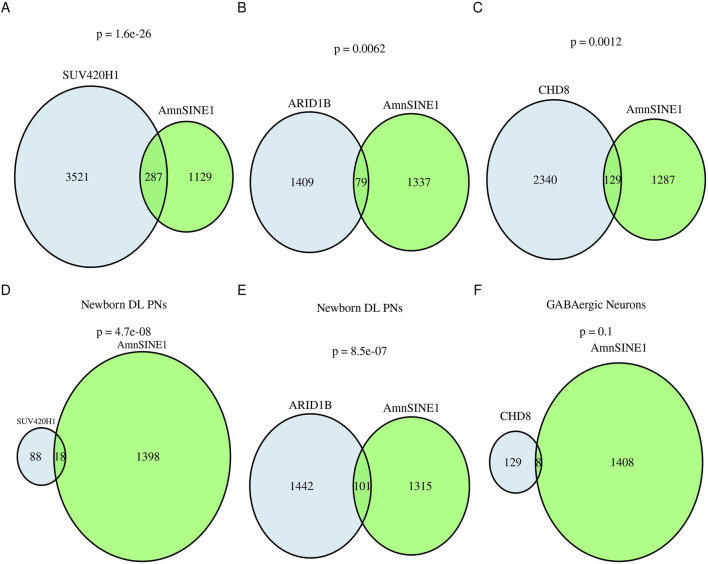
AmnSINE1-containing genes in brain organoids. Venn diagrams representing the intersection of AmnSINE1-containing loci (in green) with DEGs in organoid models of the human cerebral cortex of ASD patients in comparison with controls (in blue) in **(A)** the *SUV420H1* mutant model, **(B)** the *ARIB1D* mutant model, and **(C)** the *CHD8* mutant model. **(D)** Newborn deep layer projection neurons in the *SUV420H1* mutant model; **(E)** Newborn deep layer projection neurons in the *ARIB1D* mutant model; and **(F)** GABAergic neurons in the *CHD8* mutant model. The hypergeometric test p-value results are reported for each intersection.

To validate the role of AmnSINE1 in autism-related disorders, we used single-cell data obtained from the work of Paulsen and colleagues (Paulsen et al., 2022). In this work, the authors used ASD organoid models of the human cerebral cortex. The authors evaluated the effects of haploinsufficiency of three ASD risk genes, *ARID1B*, *CHD8* and *SUV420H1,* using single-cell RNA sequencing (scRNA-seq). We found that the genes differentially expressed in these three models compared with those in the controls significantly overlapped (hypergeometric test: ARID1B, p-value = 0.0062; SUV420H1, p-value = 1.6 ⨯ 10-26; CHD8, p-value = 0.0012) with the AmnSINE1-containing genes (Figures 4A–C). As expected, a disease enrichment analysis showed that these genes are enriched in mental disorders such as ASD and intellectual disability (Supplementary Table S7).

By analyzing the cell-type subpopulation affected by asynchronous development due to the three mutations, we found that there was a significant intersection of newborn deep-layer excitatory projection neurons at the first month (hypergeometric test *ARID1B* p-value = 8.5 ⨯ 10^−07^, *SUV420H1* p-value = 4.7 ⨯ 10^−8^) but not a significant intersection with GABAergic neurons at the third month (hypergeometric test *CHD8* p-value = 0.1) (Figures 4D–F).

### Competing endogenous RNA analysis of AmnSINE1 suggests that AmnSINE1 RNAs can act as trans-regulatory element

**FIGURE 5:**
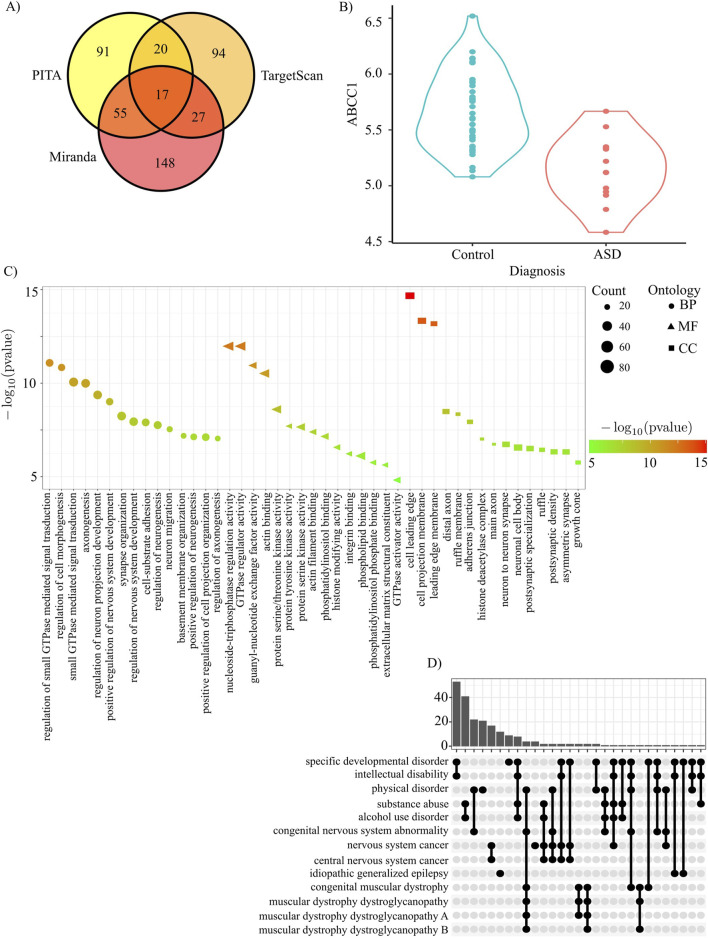
AmnSINE1 target genes identified *via* ceRNA analysis. **(A)** A Venn diagram was constructed to show the intersection of the miRNAs predicted by the three different algorithms: PITA, Miranda, and TargetScan. **(B)** ABCC1 is significantly downregulated in ASD (adjusted p-value = 0.0018). **(C)** Gene Ontology results in terms of biological processes, molecular functions and cellular components. The first 15 significant terms are represented. **(D)** Disease Ontology enrichment analysis results.

The ceRNA hypothesis states that RNAs can regulate other transcripts by competing for shared miRNAs. Leveraging three different algorithms for miRNA prediction, we found that they all agreed on 17 miRNAs: hsa-miR-216a-3p, hsa-miR-34b-5p, hsa-miR-769-3p, hsa-miR-4313, hsa-miR-3681-3p, hsa-miR-4436a, hsa-miR-4701-5p, hsa-miR-371b-3p, hsa-miR-5195-5p, hsa-miR-6718-5p, hsa-miR-6790-3p, hsa-miR-6806-5p, hsa-miR-6812-5p, hsa-miR-6831-5p, hsa-miR-6873-5p, hsa-miR-7155-5p, hsa-miR-7156-3p (Figure 5A). Putative ceRNAs (i.e., the genes predicted to be regulated by the same miRNAs that regulate AmnSINE1) were identified through the MBS database (Arancio et al., 2023) (Supplementary Table S3). The sequence that showed the greatest intersection with AmnSINE1 was *SNHG14*. Interestingly, *SNHG14* is transcribed into a long noncoding RNA that hosts SNORD116, whose loss contributes to Prader-Willi syndrome (PWS) etiology (Ariyanfar and Good, 2022); indeed, many individuals affected by PWS also have cooccurring ASD (Dykens et al., 2011).

The 2001 genes regulated by at least 5 miRNAs were subjected to gene ontology and gene pathway enrichment analyses. Gene ontology analysis clearly highlighted that the putative ceRNAs of AmnSINE1 are enriched in several nervous system- and neuron-related terms (Figure 5C). Disease enrichment analysis revealed that these genes are also primarily enriched in developmental disorders and intellectual disability (Figure 5D). Complete results of gene ontology and disease enrichment analysis are reported in Supplementary Table S8. Among the putative ceRNAs regulated by at least 5 miRNAs, only *ABCC1* is significantly downregulated in the DLPFC ASD sample (Figure 5B).

The same analysis performed on organoid datasets yielded a more complex scenario showing that differentially expressed genes are enriched in terms related to neurogenesis in all the three organoid models under investigation (Supplementary Figure S2; Supplementary Table S9).

## Discussion

Most transcriptome studies on ASD have focused on protein-coding genes. Therefore, studies on the role of the RSs in individuals with ASD have been limited. One exception is represented by the study of Saeliw and colleagues (Saeliw et al., 2023), which reported a role for Alu elements in ASD. The authors characterized the expression profiles of a portion of RSs (i.e., the transposable elements omitting sequences such as satellite repeats) in the prefrontal cortex tissues of ASD patients and controls using RNA-sequencing data. Moreover, they used the dataset from Liu et al. (2016) that is realized analyzing an RNA-seq library obtained by using poly-A enrichment and not by the use of ribodepletion, therefore a significant portion of RSs (i.e., the non-poly-adenylated RS transcripts) is lost during the library preparation. Overall, their interesting and elegant results pointed to the evident involvement of Alu sequences in ASD, but these findings lack indeed the vast contribution of non-polyadenylated transcripts.

In this work, to test whether RSs may play a role in ASD, we investigated RS transcription in the *postmortem* DLPFC of ASD patients and matched controls from the ribodepleted RNA-seq library of Wright et al. (2017) using a validated pipeline (Arancio, 2019). This pipeline allows us to investigate the expression of all classes of RSs (i.e., human endogenous retroviruses, DNA transposons, satellite repeats, LINEs and SINEs) organized in approximately one thousand RS consensus representative sequences.

Among the canonical genes and the RSs, only 42 were differentially expressed between ASD patients and controls. Even if the analysis could retrieve the expression of all the classes of RSs, strikingly, only one RS was differentially expressed between ASD patients and controls: the SINE AmnSINE1. Notably, AmnSINE1 was one of the most highly expressed genes among the differentially expressed genes, and none of the other differentially expressed genes contained this sequence. Indeed, the genes containing AmnSINE1 were significantly more highly expressed than those not containing this RS. This could be due to expression of the AmnSINE1 gene or to a generalized association between AmnSINE1 and highly expressed loci. We evaluated the expression of RSs in other brain areas of ASD patients (i.e., corpus callosum or cerebellar Purkinje cell) but we retrieved that AmnSINE1 is differentially expressed specifically in DLPFC but not in the other areas investigated (Supplementary Table S4; Supplementary Figure S1), suggesting that AmnSINE1 may play a crucial role in differentiating DLPFC of ASD patients in comparison with healthy controls. Noteworthy, DLPFC is an area crucial for executive functions, such as cognitive flexibility, that are specifically affected in ASD patients (Moxon-Emre et al., 2021).

Overall, compared with healthy controls, ASD patients had approximately twofold lower expression of AmnSINE1. Therefore, our analysis focused on the possible role of AmnSINE1 in ASD.

Literature about AmnSINE1 and its role in human disease is sporadic at best. To date, Pubmed research on AmnSINE1 was conducted, and only 7 entries were retrieved. AmnSINE1 is the short form of the ‘Amniota SINE1’, and it is part of an ancient SINE family that was first identified 310 million years ago. Its structure is a chimera of sequences similar to those of 5S rRNA and tRNA. Notably, many AmnSINE1 loci in humans are phylogenetically highly conserved among mammalian orthologs, strongly suggesting that they have acquired genomic functionality (Nishihara et al., 2006). AmnSINE1 elements are usually located in gene-poor regions in many mammalian species. Therefore, its distribution is different from that of other SINEs, such as Alu elements, which are usually located in GC-rich and gene-rich regions in genomes (Hirakawa et al., 2009). This strikingly resembles the distribution of ASD genes that are overrepresented in gene-poor regions (McGuire et al., 2016). Moreover, AmnSINE1 elements can play specific roles in the developing mammalian forebrain (Sasaki et al., 2008; Tashiro et al., 2011).

This SINE is present in approximately one thousand and half copies in the human genome, and when we investigated whether the biological activities of AmnSINE1-containing loci could be enriched in some gene ontology entries, we strikingly found that they are involved specifically in axon development and neuron differentiation and are also enriched in neuron-specific cellular components. Moreover, a disease enrichment analysis showed that the aforementioned loci are enriched in mental-related disorders, including autism spectrum disorders.

To confirm that AmnSINE1 can be directly involved in ASD in the developing human brain, we used the SFARI gene database, which is a database for the autism research community that is centered on genes implicated in autism susceptibility. The intersection between AmnSINE1-containing loci and SFARI genes was highly significant, highlighting that AmnSINE1-containing loci and genes implicated in autism susceptibility were not randomly interlaced. Notably, 40 shared genes are considered syndromic genes. Among them, there are genes that are studied in depth for their involvement in ASD, such as *CNTNAP2* (contactin-associated protein-like 2) (St George-Hyslop et al., 2022), whose gene products are important for neuronal development and synapse formation and are associated with multiple neurodevelopmental disorders, including ASD; *mTOR* (mechanistic target of rapamycin) (Thomas et al., 2023; Vasic et al., 2021), involved in aberrant neuronal development; and *RORA* (RAR-related orphan receptor A) (Sarachana and Hu, 2013), which codes for a sex hormone-sensitive nuclear receptor active in the brain that regulates the expression of many ASD-relevant genes.

The involvement of AmnSINE1 in ASD is supported by the analysis of the network of TFs involved in the regulation of transcription of genes that reside nearby AmnSINE1 sequences. The most striking evidence obtained by this analysis is the presence of many HOX genes. They are a well-known group of genes that play a fundamental role in development and segmentation during embryonic development, including the formation of the nervous system (Gonçalves et al., 2020). Evidence suggests that subtle alterations in HOX gene function may contribute to the neurodevelopmental abnormalities observed in ASD. For example, mutations or polymorphisms in genes such as *HOXA1* have been linked to alterations in brainstem development and ASD (Ingram et al., 2000). However, HOX gene involvement in ASD is still controversial (Song et al., 2011). The possible involvement of the network of stemness-specific genes, such as *SOX2* and *POU5F1*, and related genes in development is also suggested. These genes indeed encode critical transcription factors involved in the regulation of gene expression, which play significant roles in embryonic development and stem cell pluripotency (Rizzino and Wuebben, 2016). Given that ASD is a developmental disorder, the correlation between AmnSINE1 and development-specific genes is notable. Taken together, these findings strongly suggest that AmnSINE1-related genes are transcriptionally coregulated and involved not only in brain formation but also specifically in ASD development.

Since ASD is a multifaceted disorder, it is very difficult to determine whether the evidence gathered here can be generalized. Using ASD organoid models of the human cerebral cortex harboring different causative mutations, we report that, in this experimental setting, the genes that are differentially expressed in ASD models significantly intersect with AmnSINE1-containing genes. Disease enrichment analysis revealed that these genes are enriched in autism spectrum disorders in patients with *ARID1B* mutations, while there is a more general enrichment in intellectual disability in patients with *SUV420H1* and *CDH8* mutations. We speculate that AmnSINE1 plays a mediating role in this risk gene. Moreover, there was a significant overlap of AmnSINE1-containing genes with genes differentially expressed in newborn deep-layer projection neurons in the first month, while there was no significant overlap with GABAergic neurons in the third month, possibly indicating that AmnSINE1 was not temporally or developmentally related.

To investigate whether AmnSINE1 transcripts can act *per se* as noncoding RNAs in ASD, we investigated how they can alter the miRNA regulatory network.

Seventeen miRNAs were predicted to regulate AmnSINE1, and those miRNAs were used to retrieve putative genes coregulated by the same miRNAs.

The gene that showed the greatest intersection with AmnSINE1 was *SNHG14. SNHG14* is involved in Prader-Willi syndrome (Ariyanfar and Good, 2022), which is often associated with ASD (Dykens et al., 2011). Examining in detail other genes that strongly intersect with the miRNAs that regulate AmnSINE1, *OBSCN* was found to be strongly related to ASD (Matta et al., 2022), even if its canonical biological role is far from brain development (Guardia et al., 2021). Another gene that was identified by the analysis was *CACNA1A,* which encodes a subunit of voltage-gated calcium channels known to be associated with ASD (Liao and Li, 2020).

Among the putative ceRNAs, only *ABCC1* is significantly downregulated in the ASD sample. This behavior is coherent with ceRNA hypothesis, i.e., the expression of ceRNA genes and AmnSINE1 should be positively correlated. Interestingly, *ABCC1* locus is within a region where deletions have been associated with ASD even if *ABCC1 per se* is not present in SFARI database (Pinto et al., 2014; Tropeano et al., 2013).

More generally, gene ontology analysis revealed that the putative ceRNAs of AmnSINE1 are enriched in several nervous system- and neuronal development-related terms (Figure 5C). Disease enrichment analysis confirmed that the ceRNA genes are also enriched in developmental disorders and intellectual disability (Figure 5D).

Overall, genes lacking AmnSINE1 exhibit significantly lower expression levels compared to those harboring AmnSINE1 across all investigated brain regions, and their expression remains unaffected by diagnostic status. However, we observed that downregulation of AmnSINE1 is a highly specific event in the DLPFC of ASD subjects. Collectively, these data suggest that the altered expression of AmnSINE1 in the DLPFC likely reflects specific changes in RNA stability or turnover in this area rather than differential expression of genes containing AmnSINE1. Therefore, the potential link between AmnSINE1 dysregulation and ASD may arise from a trans-regulatory effect of the AmnSINE1 RNA, rather than a cis-regulatory influence of the AmnSINE1 locus on adjacent genes.

## Conclusion

AmnSINE1 transcription is significantly and specifically downregulated in ASD specimens compared with that in controls. A comprehensive investigation of the human genetic loci that contain AmnSINE1 has revealed a strong association between the genes located at these loci and both nervous system development and autism susceptibility. Furthermore, AmnSINE1 transcripts could alter the miRNA regulatory landscape for genes involved in neurogenesis and nervous system development.

Overall, AmnSINE1 emerges as a compelling new candidate that may play a role in the development of ASD.

## Data Availability

Publicly available datasets were analyzed in this study. These datasets can be downloaded from the links below: https://www.ebi.ac.uk/ena/browser/view/PRJNA398545; https://www.ebi.ac.uk/ena/browser/view/PRJNA869106; https://www.ebi.ac.uk/ena/browser/view/PRJNA263196; https://singlecell.broadinstitute.org/single_cell/study/SCP1129/asd-mutated-brain-organoids.

## Supplementary material

SUPPLEMENTARY TABLE S1 Genomic loci that contain AmnSINE1.

SUPPLEMENTARY TABLE S2 Predicted miRNAs that can bind AmnSINE1 transcripts.

SUPPLEMENTARY TABLE S3 Putative ceRNAs identified through the MBS database.

SUPPLEMENTARY TABLE S4 Differential expression analysis in dorsolateral prefrontal cortex, corpus callosum, and Purkinje cells.

SUPPLEMENTARY TABLE S5 Gene Ontology and disease enrichment analysis of genomic loci that contain AmnSINE1.

SUPPLEMENTARY TABLE S6 Transcription factors for which the promoter regions of AmnSINE1 genes are enriched in corresponding binding sites.

SUPPLEMENTARY TABLE S7 Gene Ontology and disease enrichment analysis of organoid models.

SUPPLEMENTARY TABLE S8 Gene Ontology and disease enrichment analysis of AmnSINE1 ceRNAs.

SUPPLEMENTARY TABLE S9 Gene Ontology and disease enrichment analysis of AmnSINE1 ceRNAs of organoid models.

SUPPLEMENTARY FIGURE S1 Violin plot of AmnSINE1 expression and Empirical cumulative distribution function (ECDF) of the expression of genes containing and not containing (dashed) the AmnSINE1 sequence in corpus callosum and Purkinje cells of ASD and control specimens.

SUPPLEMENTARY FIGURE S2 Volcano plots of ceRNAs for organoid models.

## Abbreviations

ASD, autism spectrum disorder; ceRNA, competing endogenous RNA; DLPFC, postmortem dorsolateral prefrontal cortex; ENA, European Nucleotide Archive; HC, Healthy control; HERV, human endogenous retrovirus; LINE, long interspersed nuclear element; LTR, long terminal repeat; miRNA, MicroRNA; PWS, Prader–Willi syndrome; RS, DNA repetitive sequence; SCARNA, small Cajal body-specific RNA; SINE, small interspersed nuclear element; SVA, SINE-VNTR-Alu; TF, transcription factor.
