# Supplementary figures and images for: Transcripts derived from AmnSINE1 repetitive sequences are depleted in the cortex of autism spectrum disorder patients
Source: Front Bioinform. 2025 Apr 9;5:1532981. doi: 10.3389/fbinf.2025.1532981 (PMC12015672; doi:10.3389/fbinf.2025.1532981)

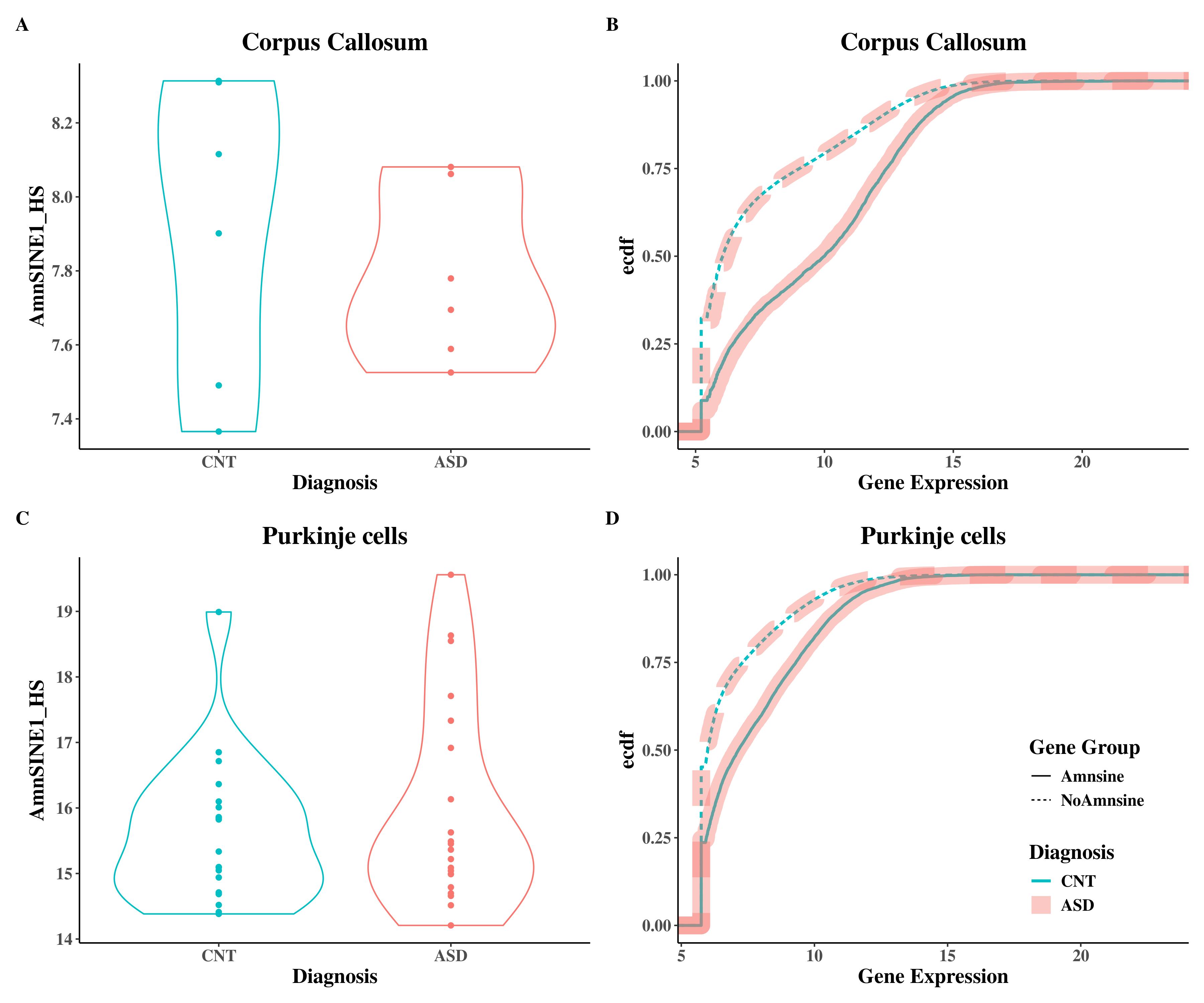

Supplement: Supplementary file 1 [file DataSheet1.zip › new_supplementary/FIGURE_S1.jpeg]

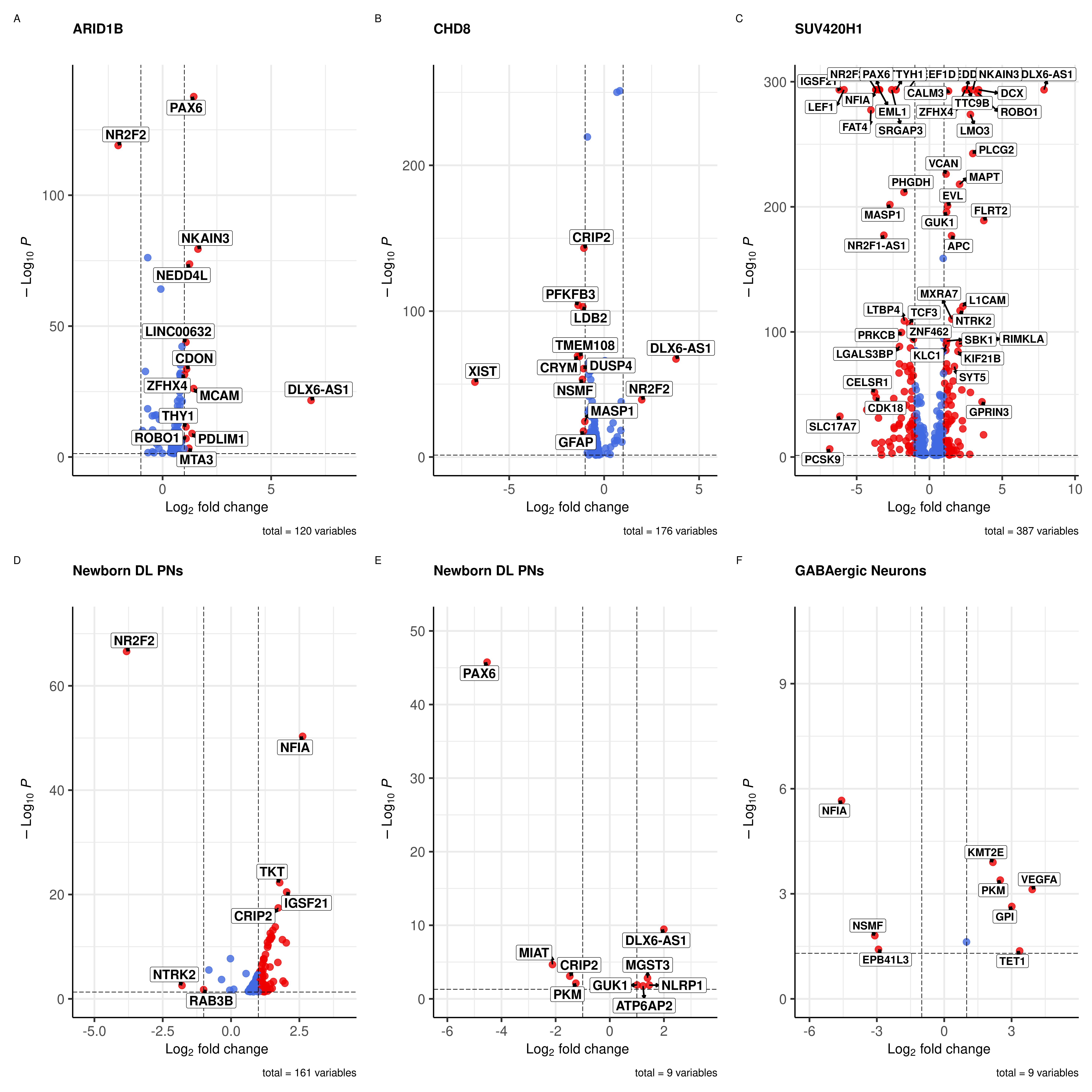

Supplement: Supplementary file 1 [file DataSheet1.zip › new_supplementary/FIGURE_S2.jpeg]
